# Multidrug-resistant enterobacterales carrying the *acc(6’)-Ian* gene

**DOI:** 10.17843/rpmesp.2026.432.15661

**Published:** 2026-06-22

**Authors:** Kristel G. Soto-Flores, Jimena R. Cisneros-Aguilar, Maricecy K. Rueda-Vicente, Edgar Gonzales-Escalante, Arturo O. Gonzales-Rodríguez, Juan C. Gomez de La Torre-Prettel, Luis A. Alvarado Ríos

**Affiliations:** 1 Universidad de Piura, Lima, Peru.; 2 Laboratorio ROE, Lima, Peru.

**Keywords:** Enterobacteriaceae, Drug Resistance, Microbial, Amikacin, Peru

## Abstract

This study describes carbapenemase-producing enterobacterales isolates obtained from urine samples collected at a private laboratory in Lima, Peru. Eighty-four carbapenem-resistant strains of *Klebsiella pneumoniae* and *Escherichia coli* were analyzed, among which 13 carried the *aac(6’)-Ian* gene. These isolates showed resistance to amikacin and other aminoglycosides. Furthermore, all isolates carried the *bla*_NDM_, *bla*_PER-2_, and *qnrVC1* genes, associated with IncC2-type plasmids, suggesting a genetic environment with high horizontal transfer potential. Through conjugation assays, we confirmed plasmid transfer to recipient strains, and through genomic analyses, we verified the co-occurrence of resistance genes and mobile genetic elements, highlighting the role of plasmids in the dissemination of these determinants in clinical settings. These findings highlight the need to implement surveillance measures to reduce the risk of infections by multidrug-resistant bacteria in the hospital setting.

## INTRODUCTION

The increasing frequency of urinary tract infections (UTI) caused by carbapenemase-producing enterobacterales (CPE), mainly *Escherichia coli* and *Klebsiella pneumoniae*, represents a public health problem due to limited therapeutic efficacy ^1^^,^^2^. The most prevalent carbapenemases, such as KPC, NDM, and OXA-48-like, spread mainly through conjugative plasmids belonging to different incompatibility groups ^3^^,^^4^.

Although β-lactams constitute the first-line therapy in UTI, the increase in resistance has maintained aminoglycosides as an alternative in infections caused by CPE^5^. In enterobacterales, the main mechanism of aminoglycoside resistance is the production of modifying enzymes (AME) ^6^. Among them, the aminoglycoside 6’-N-acetyltransferase AAC(6’)-Ian, encoded by *aac(6’)-Ian*, has emerged as a determinant of amikacin resistance ^5^^,^^7^; however, its circulation in Latin America and Peru is poorly known.

In the national context, the presence of IncC2 plasmids associated with *bla*_*NDM-1*_ and other resistance determinants in enterobacterales has been described ^8^, which suggests the existence of genetic platforms capable of concentrating and transferring multiple resistance genes. In this scenario, it is relevant to evaluate the association between *aac(6’)-Ian* and IncC2 plasmids, given their possible implication in the simultaneous dissemination of carbapenem and aminoglycoside resistance.

The present study aimed to characterize amikacin-resistant carbapenemase-producing Enterobacterales carrying *aac(6’)-Ian*, evaluating their resistance profile, genomic context, and association with IncC2 plasmids in urinary isolates from Lima, Peru.

**KEY MESSAGES**

Motivation for the study. Amikacin resistance in urinary tract infections represents a growing challenge due to the decrease in available therapeutic options against carbapenem-resistant strains. In this study, strains of *E. coli* and *K. pneumoniae* carrying the *aac(6’)-Ian* gene, associated with amikacin resistance, were identified.

Main findings. The *aac(6’)-Ian* gene was detected in 13 of 84 analyzed urinary samples, in association with other genes that favor its spread.

Public health implications. Our results underscore the urgency of strengthening microbiological surveillance and the rational use of antibiotics to prevent increasingly difficult-to-treat infections.

## THE STUDY

### Study design

An observational, descriptive, cross-sectional study was conducted aimed at the microbiological and molecular characterization of amikacin-resistant CPE.

### Population and data source

The population consisted of 56 *K. pneumoniae* and 28 *E. coli* carbapenemase-producing isolates, obtained from urine samples collected between January 2020 and December 2022 at a single branch of a private laboratory in the Lima Centro area, Peru, and sent to the Laboratorio de Microbiologia e Inmunologia de la Universidad de Piura.

### Criteria for selection

Isolates of CPE with reduced susceptibility to amikacin (minimum inhibitory concentration (MIC) ≤ 8) were included.

### Procedures

Bacterial identification was performed using the VITEK®2 system (bioMérieux, Marcy-l’Étoile, France). The susceptibility profile was evaluated using the disk diffusion method following the guidelines established in document M02-14, 24th edition of the Clinical and Laboratory Standards Institute ^9^^).^ Carbapenemase production was confirmed using the Blue Carba method. The complete nucleotide sequences were deposited in GenBank under accession number PRJNA1220724.

The antibiotics evaluated by disk diffusion included: ciprofloxacin (CIP) 5μg, gentamicin (CN) 10μg, amikacin (AK) 30μg, ceftazidime (CAZ) 30μg, aztreonam (ATM) 30μg, trimethoprim/sulfamethoxazole (SXT) 1.25/23.75μg, cefotaxime (CTX) 30μg, meropenem (MEM) 10μg, imipenem (IPM) 10μg, ceftazidime/clavulanic acid (CZC) 30/10μg, ertapenem (ETP) 10μg, piperacillin/tazobactam (TZP) 100/10μg, cefoxitin (FOX) 30μg and ceftazidime/avibactam (CZA) 10/4μg.

The MIC of the isolates against amikacin, gentamicin, and kanamycin was determined with dilution ranges of 0.25, 0.5, 1, 2, 4, 8, 16, 32, 64, and 128 μg/mL. For plazomicin, it was established by epsilometry. *E. coli* ATCC 25922 was used as a control strain ^8^.

We used endpoint PCR to specifically detect *aac(6’)-Ian* and determine its frequency in the isolates, following a standardized *in-house* protocol with primers designed in this study (table 1) and amplification conditions described in the supplementary material.

**Table 1:** Primers used in PCR amplification.

Primers	Objective	Base pairs (bp)	Melting temperature	Reference
5’-TACTTCCTATACTCAGCAATTTCGG-3’ Rv: 5’-CTTCATGCATTTCATGGGCG-3’	*aac(6’)-Ian*	583	72 °C	This study
ATGAATGTCATCACAAAATGTGT Rv: TCAATCCGGACTCACTGCAG	*bla*_*PER-2*_	927	52 °C	Gonzales Escalante ^8^^)​^
Fw: 5’-GGTTTGGCGATCTGGTTTTC-3’ Rev: 5’-CGGAATGGCTCATCACGATC-3’	*bla*_*NDM*_	621	52 ºC	Gonzales Escalante ^8^^)​^
Fw: 5’-TTAGTCAGGAACAATGATTAC-3’ Reverse: 5’-TGGAAAAATCAAAGCAATTAT-3’	*qnrVC1*	656	45 ºC	Rincón ^10^
Fw: 5’- CGCTGCCGAAGTACATTTGG-3’ Rv: 5’- TTGCCCTCGATGATATGGCC-3’	IncC2	282	51 °C	This study

Fw: Forward primer (forward primer), Rv: Reverse primer (reverse primer).

In positive isolates, *qnrVC1, bla*_NDM_ and *bla*_PER-2_ were sought using standardized PCR protocols described by Rincón ^10^ and Gonzales Rodriguez ^8^, respectively. Additionally, the IncC2 replicon was detected using an *in-house* PCR protocol based on primers designed in this study (supplementary material) (table 1). In both procedures, we used *E. coli* Col16 (GenBank: JANIDU000000000) as a positive control and *K. pneumoniae* Col17 (GenBank: CP072905) as a negative control. Short reads (NextSeq, Illumina Paired-end PE) and long reads (MinION, Oxford Nanopore Technologies) were combined for sequencing.

We performed the conjugation assay according to the methodology described by Gonzales-Rodriguez *et al*^11^. Each isolate carrying the *aac(6’)-Ian* gene was used as a donor strain, and azide-resistant *E. coli* J53 (EcJ53) was used as the recipient strain, in a 1:1 ratio for 18 hours at 37 °C.

To evaluate plasmid mobilization, we used EcJ53, selected in a medium containing sodium azide (150 μg/mL), amikacin (50 μg/mL) and ampicillin (50 μg/mL), with a second selection in a medium with sodium azide (150 μg/mL), amikacin (50 μg/mL) and imipenem (5 μg/mL). We confirmed the presence of the genes *aac(6’)-Ian*, *qnrVC1*, *bla*_NDM_, *bla*_PER-2_ and of the IncC2 replicon in the transconjugant strains by endpoint PCR.

We extracted the genomic DNA with the Genomic Purification kit (Thermo Fisher Scientific, USA) and prepared the *paired-end* libraries with the Illumina Nextera XT kit, following the manufacturer's instructions in both cases.

### Data analysis

Six isolates randomly selected from among the 13 strains carrying the *aac(6’)-Ian* gene and the IncC2 plasmid were analyzed by sequencing using Illumina MiSeq with 2 × 250 bp *paired-end* reads. We filtered low-quality reads (Phred scores <30) using Trimmomatic v0.39. We performed de novo genome assembly with Unicycler v0.4.8 and annotated the assembled genomes with Prokka v1.14.6, complementing with manual curation. We identified sequence types (ST) using MLSTfinder v2.0, resistance genes with ResFinder v1, and plasmid incompatibility groups with PlasmidFinder v2.1.

### Ethical considerations

The Institutional Research Ethics Committee of the Universidad de Piura approved the protocol for this study (PREMED06202330). A material transfer agreement was obtained for the use of the samples.

## FINDINGS

Of 84 strains with reduced susceptibility to amikacin, 13 carriers of *aac(6’)-Ian* (8 *K. pneumoniae*, 5 *E. coli*) were identified. Antimicrobial susceptibility tests revealed resistance to all β-lactamase inhibitors and to sulfamethoxazole/trimethoprim, with preserved susceptibility to gentamicin and intermediate susceptibility to ciprofloxacin. All isolates carried *bla*_NDM_ (13/13) and *qnrVC1* (13/13) and were positive for the IncC2 replicon (13/13), with the exception of *bla*_PER-2_ which was found in 12/13 isolates (table 2).

**Table 2:** Distribution of genotypic co-resistance profiles in carbapenemase-producing Enterobacterales (CPE) isolates carrying the *aac(6’)-Ian* gene (n=13).

Resistance profile	***E. coli* (n=5)**	***K. pneumoniae* (n=8)**	Total (N=13)^a^	Frequency (%)
*aac(6')-Ian* + *bla*_*NDM*_ + *bla*_*PER-2*_ + *qnrVC1*	5	7	12	92.3
*aac(6')-Ian* + *bla*_*NDM*_ + *qnrVC1*	0	1	1	7.7
Total	5	8	13	100.0

a Among 84 amikacin-resistant isolates, 13 were positive for the presence of the gene *aac(6’)-Ian*.

The plasmid carrying *aac(6’)-Ian* was successfully transferred from the 13 wild strains to *E. coli* J53, obtaining 13 transconjugants carrying *bla*_NDM_, *bla*_PER-2_, and *qnrVC1*. The MICs of the transconjugants showed high levels of resistance to amikacin and kanamycin, while retaining susceptibility to gentamicin and plazomicin (table 3).

**Table 3:** Antibiotic susceptibility of carbapenemase-producing enterobacterial isolates (CPE) carrying *aac(6’)-Ian* and their transconjugants.

Isolates	MIC (μg/mL)			
	Kanamycin	Amikacin	Gentamicin	Plazomicina
K1	512	32	64	1
TC K 1	64	32	Please provide the Spanish text you wish to translate.	0.5
K8	512	32	128	1
TC K8	256	32	1	0.38
K82	64	16	0.5	0.5
TC K82	256	16	1	0.5
K83	64	16	0.5	0.75
TC K83	256	32	1	0.38
K93	64	16	0.5	0.75
TC K93	512	64	0.5	0.5
K101	128	32	0.5	0.38
TC K101	128	64	0.5	0.38
K118	128	32	1	0.5
TC K118	128	64	0.5	0.38
K367	512	32	0.5	0.75
CT K367	128	32	0.5	0.5
EC18	512	128	128	1
TC EC18	512	64	0.5	0.5
EC260	256	128	1	1.5
TC EC260	128	64	0.5	0.5
EC306	512	256	1	1
TC EC306	128	64	0.5	0.38
EC342	256	128	64	0.75
TC EC342	128	64	0.5	0.19
EC361	256	64	0.5	0.75
TC EC361	128	64	0.5	0.75

MIC: Minimum inhibitory concentration, TC: Transconjugants, K: *K. pneumoniae*, EC: *E. coli*.

Genomic sequencing of six randomly selected isolates revealed different sequence types (ST) for *K. pneumoniae* ST629 and ST13, while *E. coli* showed greater diversity: ST219, ST167, ST131 and ST4628.

A variety of antimicrobial resistance genes were identified coexisting in the same genetic environment, among them those associated with aminoglycosides [*aac(3)-IIa, aac(6’)-Ian, aph(6)-Id, aph(3’’)-Ib, aph(3’)-Ia, aadA, aadA2*], quinolones [*qnrVC1, aac(6’)-Ib-cr, qnrB1, qnrS1*], phenicols [*cmlB1, catB3, arr-3, catA2, catII*], streptomycin [*strA, strB*], β-lactams [*bla*_*NDM-1*_ *, bla*_*PER-2*_ *, bla*_*SHV-145*_ *, bla*_*CMY-136*_ *, bla*_*TEM-1B*_ *, bla*_*OXA-1*_ ], trimethoprim [*dfrA12, dfrA6, dfrA14*], fosfomycin [*fosA6*], sulfamethoxazole [*sul1, sul2, sul3*], and macrolides [*mph(A)* ]. Additionally, the following replicons were identified: IncFII, IncFIA, IncFIB, IncX, IncR, colRNAI, and IncC2.

Genomic alignment of the sequenced isolates was performed against pEcCol16ndm (GenBank: NZ_JANIDU010000010.1), previously recovered from a blood sample of an oncological patient in Peru in 2017 and characterized by carrying *aac(6’)-Ian, bla*_*NDM-1*_ *, bla*_*PER-2*_ *and qnrVC1*^8^. In our genomic set, a coverage level greater than 95% was identified in all alignments. Likewise, in some *contigs* of the sequenced genomes, the IncC2 replication origin was observed within the same genetic environment as *aac(6’)-Ian, bla*_*NDM-1*_ *, bla*_*PER-2*_ *and qnrVC1*. In some genomes (EC361, KP1, EC306 and EC18), it was possible to establish the genetic environment surrounding *aac(6’)-Ian*, configured by: [IS26-floR-ISAba43-IS1006-IS91-aac(6’)-Ian-ΔIS1006-ISVsa3].

## DISCUSSION

This study confirms the presence of *aac(6’)-Ian* in 15.5% (13/84) of *K. pneumoniae* and *E. coli* isolates with reduced susceptibility to amikacin, associated with bla_NDM_, *bla*_*PER-2*_ , and *qnrVC1* genes. These determinants are located on highly transferable IncC2 plasmids, whose genomic structure maintains an identity greater than 95% with the *pEcCol16ndm* plasmid previously detected in Peru ^8^, demonstrating the persistence of a multi-drug resistance platform in the region.

The coexistence of *bla*_*NDM*_ *, bla* ^*PER-2*^ *and qnrVC1* previously described in Peru in an IncC2 replicon ^8^ was also identified in wild-type strains and their transconjugants, which explains resistance to amikacin, kanamycin, and sulfamethoxazole, associated with resistance to β-lactams, β-lactamase inhibitors, and decreased susceptibility to quinolones, without affecting gentamicin activity.

Furthermore, the analysis of the 13 CPEs confirmed the presence of the IncC2 replicon. The *aac(6’)-Ian* gene has been previously reported in this type of mobilizable plasmids and has been associated with *bla*_*NDM-1*_ *, bla*_*CTX-M-15*_ *, bla*_*GES-2*_ *, bla*_*TEM-1A*_ *, blaOXA-1, bla*_*ACT-15*_ *, aac(3’)-IIa*^12^*, as well as with bla*_*TLA-3*_ *, bla*_*TEM*_ and *aac(3’)-II*^7^.

We corroborated these findings by genomic sequencing of six *aac(6’)-Ian*-carrying strains selected by convenience. Gonzales Escalante reported a genetic configuration surrounding *aac(6’)-Ian* that included *qnrVC1*, *bla*_*NDM*_ and *bla*_*PER-2*_ , embedded in the ARI-B resistance islands in an *E. coli* strain ^8^. Furthermore, the alignment analysis with NZ_JANIDU010000010.1 showed extensive sequence coverage, which supports the hypothesis that the IncC2 plasmid has been circulating in the local environment for at least five years ^8^. The presence of the IncC2 type disseminated in various STs allows us to infer its successful spread.

Plazomicin activity was not affected by the presence of *aac(6’)-Ian*, similar to gentamicin; this suggests that both could be therapeutic options against CPEs producing this enzyme. The preservation of plazomicin activity could be explained by structural differences, such as the 2-hydroxyethyl group at position 6’, which would allow it to evade this resistance mechanism ^13^. Although these *in vitro* results are encouraging, their clinical implementation will depend on their availability in local settings; therefore, additional studies are required to consolidate their usefulness against carbapenemase-associated multidrug-resistant strains.

In Latin America, the circulation of plasmids accumulating multiple resistance determinants has been documented. Millán *et al*. demonstrated the mobilization of *bla*_*CTX-M*_ associated with ISCR1 in IncN and IncFIIA plasmids in Venezuela, while regional reports show the co-circulation of ESBLs and carbapenemases in hospital settings ^14^. Recently, Cordeiro *et al*. described *bla*_*NDM-1*_ in an IncC2 plasmid in Uruguay, highlighting the hemispheric emergence of this platform^15^. In this context, the association of *bla*_*NDM*_ with IncC2 observed in our study is integrated into a regional pattern of multiresistant plasmid evolution that limits therapeutic options and reinforces the need to strengthen genomic surveillance.

The growing regional detection of mechanisms such as those described in this study reinforces the need to standardize genomic sequencing within the framework of GLASS (Global Antimicrobial Resistance Surveillance System) ^16^, incorporating molecular tools into the surveillance of priority pathogens. This would strengthen clinical decision-making and the integration of Latin America into global resistance monitoring networks. At the national level, the articulation of these strategies with antimicrobial use optimization programs (PROA) is fundamental to limit the expansion of emerging plasmid platforms such as IncC2 ^17^.

This study presents limitations that must be acknowledged. The isolates came from a single private laboratory, which may affect their national representativeness, and detailed clinical information was not available. Furthermore, 13 of 84 strains were positive for *aac(6’)-Ian* and only six were sequenced, which restricts the structural generalization of the plasmid. However, these aspects do not diminish the epidemiological relevance of the findings.

In conclusion, we report the presence of *aac(6’)-Ian* in 13 amikacin-resistant CPE isolates carrying *bla*_*PER-2*_ *, bla*_*NDM*_ and *qnrVC1* on IncC2 plasmids. These findings indicate the risk of resistance dissemination among bacterial species, reducing therapeutic options for complicated UTIs due to their horizontal dissemination potential.
