# Supplementary material for: Multidrug-resistant enterobacterales carrying the acc(6’)-Ian gene
Source: Rev Peru Med Exp Salud Publica. 2026 Jun 22;43(2):238–43. doi: 10.17843/rpmesp.2026.432.15661 (PMC13456922; doi:10.17843/rpmesp.2026.432.15661)
Supplement: Supplementary file 1 [file rpmesp-43-02-15661-s001-es.docx]

**Material suplementario (Anexos)**

**Anexo 1. Protocolo y validación analítica de la PCR para la detección del gen aac(6′)-Ian**

La amplificación del gen aac(6′)-Ian se realizó mediante PCR de punto final utilizando cebadores diseñados en este estudio (Tabla 1). Los cebadores fueron diseñados a partir de secuencias del gen disponibles en bases de datos públicas, seleccionando regiones conservadas y evaluando su especificidad mediante análisis in silico, con el fin de minimizar amplificaciones cruzadas con genes homólogos.

Para un volumen final de 25 μL, utilizamos 2 μL de ADN, 1.0 μM de cada cebador, 10 mM de dNTPs, 0,5 U/L de Taq DNA Polimerasa (Cav Bio, Lima, Perú) y 2,5 μL de tampón de PCR 10X con Mg²⁺. Desnaturalizamos las muestras de ADN a 94°C durante 2 minutos, seguidos de 30 ciclos de amplificación a 94°C por 1 minuto, 55°C por 1 minuto y 72°C por 1 minuto. La elongación se realizó a 72°C durante 10 minutos para completar la extensión de los productos de PCR

El desempeño del ensayo fue evaluado previamente utilizando un panel de aislamientos positivos y negativos para el gen aac(6′)-Ian, cuya presencia o ausencia había sido confirmada mediante secuenciación genómica completa (WGS). Bajo las condiciones optimizadas, el ensayo mostró amplificación en el 100% de los aislamientos positivos y ausencia de amplificación en los controles negativos, evidenciando concordancia completa con los resultados de secuenciación.

Adicionalmente, el ensayo fue evaluado en corridas independientes, obteniéndose resultados consistentes entre replicados, lo que respalda la reproducibilidad y especificidad del método.

**Anexo 2. Protocolo de PCR para la detección del replicón plasmídico IncC2**

La detección del replicón IncC2 se realizó mediante PCR de punto final utilizando cebadores diseñados en este estudio (Tabla 1). En un volumen final de 25 μL, se utilizó 2,5 μL de ADN, 1,0 μM de cada cebador, 10 mM de dNTPs, 0,5 U/L de Taq DNA Polimerasa y 2,5 μL de tampón de PCR 10X con Mg²⁺. Iniciamos con una desnaturalización a 94°C durante 5 minutos, seguida de 30 ciclos de 94°C por 1 minuto, 51°C por 1 minuto y 72°C por 1 minuto. Finalizamos con una elongación a 72°C durante 5 minutos.

La validación analítica del ensayo se realizó siguiendo el mismo procedimiento descrito para la detección del gen aac(6′)-Ian, utilizando aislamientos previamente caracterizados y confirmando la reproducibilidad y especificidad del método.
