# Supplementary material for: Multidrug-resistant enterobacterales carrying the acc(6’)-Ian gene
Source: Rev Peru Med Exp Salud Publica. 2026 Jun 22;43(2):238–43. doi: 10.17843/rpmesp.2026.432.15661 (PMC13456922; doi:10.17843/rpmesp.2026.432.15661)
Supplement: Translation

# Translation: Enterobacterales multirresistentes portadores del gen *acc(6’)-Ian*

**Authors:** Kristel G. Soto-Flores, Jimena R. Cisneros-Aguilar, Maricecy K. Rueda-Vicente, Edgar Gonzales-Escalante, Arturo O. Gonzales-Rodríguez, Juan C. Gomez de La Torre-Prettel, Luis A. Alvarado Ríos

**Affiliations:** 1 Universidad de Piura, Lima, Perú.; 2 Laboratorio ROE, Lima, Perú.

**Keywords:** Enterobacteriaceae, Farmacorresistencia Microbiana, Amikacina, Perú

## Abstract

Este estudio describe aislamientos de enterobacterales productores de carbapenemasas obtenidos de muestras de orina recolectadas en un laboratorio privado de Lima, Perú. Se analizaron 84 cepas resistentes a carbapenémicos de *Klebsiella pneumoniae* y *Escherichia coli*, entre las cuales 13 portaban el gen *aac(6’)-Ian*. Estos aislamientos mostraron resistencia a amikacina y a otros aminoglucósidos. Asimismo, todos los aislamientos portaban los genes *bla*_NDM_, *bla*_PER-2_ y *qnrVC1*, asociados a plásmidos del tipo IncC2, lo que sugiere un entorno genético con alto potencial de transferencia horizontal. Mediante ensayos de conjugación, confirmamos la transferencia plasmídica a cepas receptoras, y a través de análisis genómicos verificamos la presencia conjunta de genes de resistencia y elementos genéticos móviles, lo que resalta el papel de los plásmidos en la diseminación de estos determinantes en entornos clínicos. Estos hallazgos resaltan la necesidad de implementar medidas de vigilancia para reducir el riesgo de infecciones por bacterias multirresistentes en el ámbito hospitalario.

## INTRODUCCIÓN

La creciente frecuencia de infecciones del tracto urinario (ITU) causadas por enterobacterales productoras de carbapenemasas (EPC), principalmente *Escherichia coli* y *Klebsiella pneumoniae*, representa un problema de salud pública debido a la limitada eficacia terapéutica ^1^^,^^2^. Las carbapenemasas más prevalentes, como KPC, NDM y OXA-48-like, se diseminan principalmente a través de plásmidos conjugativos pertenecientes a distintos grupos de incompatibilidad ^3^^,^^4^.

Aunque los β-lactámicos constituyen la primera línea terapéutica en ITU, el incremento de la resistencia ha mantenido a los aminoglucósidos como alternativa en infecciones causadas por EPC ^5^. En enterobacterales, el principal mecanismo de resistencia a aminoglucósidos es la producción de enzimas modificadoras (AME) ^6^. Entre ellas, la aminoglucósido 6’-N-acetiltransferasa AAC(6’)-Ian, codificada por *aac(6’)-Ian*, ha emergido como determinante de resistencia a amikacina ^5^^,^^7^; sin embargo, su circulación en Latinoamérica y en Perú es poco conocida.

En el contexto nacional, se ha descrito la presencia de plásmidos IncC2 asociados a *blaNDM-1* y otros determinantes de resistencia en enterobacterales ^8^, lo que sugiere la existencia de plataformas genéticas capaces de concentrar y transferir múltiples genes de resistencia. En este escenario, resulta pertinente evaluar la asociación entre *aac(6’)-Ian* y plásmidos IncC2, dada su posible implicancia en la diseminación simultánea de resistencia a carbapenémicos y aminoglucósidos.

El presente estudio tuvo como objetivo caracterizar enterobacterales productoras de carbapenemasas resistentes a amikacina portadoras de *aac(6’)-Ian*, evaluando su perfil de resistencia, su contexto genómico y su asociación con plásmidos IncC2 en aislamientos urinarios de Lima, Perú.

**MENSAJES CLAVE**

Motivación para realizar el estudio. La resistencia a amikacina en infecciones urinarias representa un desafío creciente debido a la disminución de opciones terapéuticas disponibles frente a cepas resistentes a carbapenémicos. En este estudio se identificaron cepas de *E. coli* y *K. pneumoniae* portadoras del gen *aac(6’)-Ian*, asociado con resistencia a amikacina.

Principales hallazgos. El gen *aac(6’)-Ian* fue detectado en 13 de 84 muestras urinarias analizadas, en asociación con otros genes que favorecen su propagación.

Implicancias en salud pública. Nuestros resultados subrayan la urgencia de fortalecer la vigilancia microbiológica y el uso racional de antibióticos para prevenir infecciones cada vez más difíciles de tratar.

## EL ESTUDIO

### Diseño del estudio

Se realizó un estudio observacional, descriptivo y transversal orientado a la caracterización microbiológica y molecular de EPC resistentes a amikacina.

### Población y fuente de datos

La población estuvo conformada por aislamientos de 56 *K. pneumoniae* y 28 *E. coli* productores de carbapenemasas, obtenidos de muestras de orina, recolectadas entre enero del 2020 y diciembre del 2022 en una única sede de un laboratorio privado de la zona de Lima Centro, Perú y remitidos al Laboratorio de Microbiología e Inmunología de la Universidad de Piura.

### Criterios de selección

Se incluyeron aislamientos de EPC con sensibilidad reducida a amikacina (concentración inhibitoria mínima (CIM) ≤ 8).

### Procedimientos

La identificación bacteriana se realizó mediante el sistema VITEK®2 (bioMérieux, Marcy-l’Étoile, Francia). El perfil de sensibilidad se evaluó utilizando el método de difusión en disco siguiendo las directrices establecidas en el documento M02-14, 24ª edición del Clinical and Laboratory Standards Institute ^9^^).^ La producción de carbapenemasas se confirmó mediante el método Blue Carba. Las secuencias nucleotídicas completas se depositaron en GenBank bajo el número de acceso PRJNA1220724.

Los antibióticos evaluados mediante difusión en disco incluyeron: ciprofloxacino (CIP) 5μg, gentamicina (CN) 10μg, amikacina (AK) 30μg, ceftazidima (CAZ) 30μg, aztreonam (ATM) 30μg, trimetoprima/sulfametoxazol (SXT) 1.25/23.75μg, cefotaxima (CTX) 30μg, meropenem (MEM) 10μg, imipenem (IPM) 10μg, ceftazidima/ácido clavulánico (CZC) 30/10μg, ertapenem (ETP) 10μg, piperacilina/tazobactam (TZP) 100/10μg, cefoxitina (FOX) 30μg y ceftazidima/avibactam (CZA) 10/4μg.

La CIM de los aislamientos frente a amikacina, gentamicina y kanamicina se determinó con rangos de dilución de 0,25, 0,5, 1, 2, 4, 8, 16, 32, 64 y 128 μg/mL. Para plazomicina se estableció mediante epsilometría. Se utilizó *E. coli* ATCC 25922 como cepa control ^8^.

Empleamos PCR de punto final para detectar específicamente *aac(6’)-Ian* y determinar su frecuencia en los aislamientos, siguiendo un protocolo *in-house* estandarizado con cebadores diseñados en este estudio (tabla 1) y condiciones de amplificación descritas en el material suplementario.

**Tabla 1:** Cebadores utilizados en la amplificación por PCR.

Primers	Objetivo	Pares de bases (pb)	Temperatura de melting	Referencia
Fw: 5’-TACTTCCTATACTCAGCAATTTCGG-3’ Rv: 5’-CTTCATGCATTTCATGGGCG-3’	*aac(6’)-Ian*	583	72 ºC	Este estudio
Fw: ATGAATGTCATCACAAAATGTGT Rv: TCAATCCGGACTCACTGCAG	*bla*_*PER-2*_	927	52 ºC	Gonzales Escalante ^8^^)​^
Fw: 5’-GGTTTGGCGATCTGGTTTTC-3’ Rv: 5’-CGGAATGGCTCATCACGATC-3’	*bla*_*NDM*_	621	52 ºC	Gonzales Escalante ^8^^)​^
Fw: 5’-TTAGTCAGGAACAATGATTAC-3’ Rv: 5’-TGGAAAAATCAAAGCAATTAT-3’	*qnrVC1*	656	45 ºC	Rincón ^10^
Fw: 5’- CGCTGCCGAAGTACATTTGG-3’ Rv: 5’- TTGCCCTCGATGATATGGCC-3’	IncC2	282	51 °C	Este estudio

Fw: Forward primer (cebador directo), Rv: Reverse primer (cebador reverso).

En los aislamientos positivos, se buscó *qnrVC1, bla*_*NDM*_ y *bla*_*PER-2*_ mediante protocolos PCR estandarizados descritos por Rincón ^10^ y Gonzales Rodriguez ^8^, respectivamente. Adicionalmente se detectó el replicón IncC2 utilizando un protocolo de PCR *in-house* basado en cebadores diseñados en este estudio (material suplementario) (tabla 1). En ambos procedimientos empleamos como control positivo *E. coli* Col16 (GenBank: JANIDU000000000) y como control negativo *K. pneumoniae* Col17 (GenBank: CP072905). Se combinaron lecturas cortas (NextSeq, Illumina Paired-end PE) y largas (MinION, Oxford Nanopore Technologies) para secuenciación.

Realizamos el ensayo de conjugación de acuerdo con la metodología descrita por Gonzales-Rodriguez *et al*^11^. Se utilizó como cepa donadora a cada aislamiento portador del gen *aac(6’)-Ian* y como cepa receptora a *E. coli* J53 resistente a azida de sodio (EcJ53), en una proporción 1:1 durante 18 horas a 37 °C.

Para evaluar la movilización plasmídica empleamos a EcJ53, seleccionada en un medio que contenía azida de sodio (150 μg/mL), amikacina (50 μg/mL) y ampicilina (50 μg/mL), con una segunda selección en un medio con azida de sodio (150 μg/mL), amikacina (50 μg/mL) e imipenem (5 μg/mL). Confirmamos la presencia de los genes *aac(6’)-Ian*, *qnrVC1*, *bla*_*NDM*_ *, bla*_*PER-2*_ y del replicón IncC2 en las cepas transconjugantes mediante PCR de punto final.

Extrajimos el ADN genómico con el kit Genomic Purification (Thermo Fisher Scientific, USA) y preparamos las bibliotecas *paired-end* con el kit Illumina Nextera XT, siguiendo en ambos casos las instrucciones del fabricante.

### Análisis de datos

Se analizó por secuenciación mediante lIlumina MiSeq con lecturas *paired-end* de 2 × 250 pb a 6 aislamientos seleccionados al azar entre las 13 cepas portadoras del gen *aac(6’)-Ian* y del plásmido IncC2. Filtramos las lecturas de baja calidad (puntajes Phred <30) mediante Trimmomatic v0.39. Realizamos el ensamblaje de novo de los genomas con Unicycler v0.4.8 y anotamos los genomas ensamblados con Prokka v1.14.6, complementando con una curación manual. Identificamos los tipos de secuencia (ST) mediante MLSTfinder v2.0, los genes de resistencia con ResFinder v1 y los grupos de incompatibilidad plasmídica con PlasmidFinder v2.1.

### Consideraciones éticas

El Comité Institucional de Ética en Investigación de la Universidad de Piura aprobó el protocolo de este estudio (PREMED06202330). Se obtuvo un acuerdo de transferencia de materiales para el uso de las muestras.

## HALLAZGOS

De 84 cepas con sensibilidad reducida a amikacina se identificó 13 portadoras de *aac(6’)-Ian* (8 *K. pneumoniae*, 5 *E. coli*). Las pruebas de sensibilidad antimicrobiana revelaron resistencia a todos los inhibidores de β-lactamasas y a sulfametoxazol/trimetoprim, con sensibilidad preservada a gentamicina y sensibilidad intermedia a ciprofloxacino. Todos los aislamientos portaban *bla*_*NDM*_ (13/13) y *qnrVC1* (13/13) y fueron positivos para el replicón IncC2 (13/13), con excepción de *bla*_*PER-2*_ que se encontró en 12/13 aislamientos (tabla 2).

**Tabla 2:** Distribución de perfiles de co-resistencia genotípica en aislamientos de enterobacterales productoras de carbapenemasas (EPC) portadores del gen *aac(6’)-Ian* (n=13).

Perfil de resistencia	***E. coli* (n=5)**	***K. pneumoniae* (n=8)**	Total (N=13)^a^	Frecuencia (%)
*aac(6')-Ian* + *bla*_*NDM*_ + *bla*_*PER-2*_ + *qnrVC1*	5	7	12	92,3
*aac(6')-Ian* + *bla*_*NDM*_ + *qnrVC1*	0	1	1	7,7
Total	5	8	13	100,0

a Entre 84 aislamientos resistentes a amikacina, 13 fueron positivos para la presencia del gen *aac(6’)-Ian*.

El plásmido portador de *aac(6’)-Ian* fue transferido exitosamente desde las 13 cepas silvestres a *E. coli* J53, obteniéndose 13 transconjugantes portadoras de *bla*_*NDM*_ *, bla*_*PER-2*_ y *qnrVC1*. Las CIM de los transconjugantes mostraron altos niveles de resistencia a amikacina y kanamicina, mientras que conservaron sensibilidad frente a gentamicina y plazomicina (tabla 3).

**Tabla 3:** Sensibilidad antibiótica de aislados de enterobacterales productoras de carbapenemasas (EPC) portadores de *aac(6’)-Ian* y sus transconjugantes.

Aislamientos	CIM (μg/mL)			
	Kanamicina	Amikacina	Gentamicina	Plazomicina
K1	512	32	64	1
TC K 1	64	32	1	0,5
K8	512	32	128	1
TC K8	256	32	1	0,38
K82	64	16	0,5	0,5
TC K82	256	16	1	0,5
K83	64	16	0,5	0,75
TC K83	256	32	1	0,38
K93	64	16	0,5	0,75
TC K93	>512	64	0,5	0,5
K101	128	32	0,5	0,38
TC K101	128	64	0,5	0,38
K118	128	32	1	0,5
TC K118	128	64	0,5	0,38
K367	512	32	0,5	0,75
TC K367	128	32	0,5	0,5
EC18	>512	128	128	1
TC EC18	>512	64	0,5	0,5
EC260	256	128	1	1,5
TC EC260	128	64	0,5	0,5
EC306	>512	>256	1	1
TC EC306	128	64	0,5	0,38
EC342	256	128	64	0,75
TC EC342	128	64	0,5	0,19
EC361	256	64	0,5	0,75
TC EC361	128	64	0,5	0,75

CMI: Concentración mínima inhibitoria, TC: Transconjugantes, K: *K. pneumoniae*, EC: *E. coli.*

La secuenciación genómica de seis aislamientos seleccionados al azar reveló diferentes tipos de secuenciotipos (ST) para *K. pneumoniae* ST629 y ST13, mientras que *E. coli* presentó mayor diversidad: ST219, ST167, ST131 y ST4628.

Se identificó una variedad de genes de resistencia antimicrobiana coexistiendo en un mismo entorno genético, entre ellos los asociados a aminoglucósidos [*aac(3)-IIa, aac(6’)-Ian, aph(6)-Id, aph(3’’)-Ib, aph(3’)-Ia, aadA, aadA2*], quinolonas [*qnrVC1, aac(6’)-Ib-cr, qnrB1, qnrS1*], fenólicos [*cmlB1, catB3, arr-3, catA2, catII*], estreptomicina [*strA, strB*], β-lactámicos [*bla*_*NDM-1*_ *, bla*_*PER-2*_ *, bla*_*SHV-145*_ *, bla*_*CMY-136*_ *, bla*_*TEM-1B*_ *, bla*_*OXA-1*_ ], trimetoprim [*dfrA12, dfrA6, dfrA14*], fosfomicina [*fosA6*], sulfametoxazol [*sul1, sul2, sul3*], y macrólidos [*mph(A)*]. Asimismo, se identificaron los siguientes replicones: IncFII, IncFIA, IncFIB, IncX, IncR, colRNAI e IncC2.

Se realizó la alineación genómica de los aislamientos secuenciados contra pEcCol16ndm (GenBank: NZ_JANIDU010000010.1), previamente recuperado de una muestra de sangre de un paciente oncológico en Perú en 2017 y caracterizado por portar *aac(6’)-Ian, bla*_*NDM-1*_ *, bla*_*PER-2*_ *y qnrVC1*^8^. En nuestro conjunto genómico se identificó un nivel de cobertura superior al 95% en todos los alineamientos. Asimismo, en algunos *contigs* de los genomas secuenciados se observó el origen de replicación de IncC2 dentro del mismo entorno genético que *aac(6’)-Ian, bla*_*NDM-1*_ *, bla*_*PER-2*_ y *qnrVC1*. En algunos genomas (EC361, KP1, EC306 y EC18) fue posible establecer el entorno genético que rodea a *aac(6’)-Ian*, configurado por: [IS26-floR-ISAba43-IS1006-IS91-aac(6’)-Ian-ΔIS1006-ISVsa3].

## DISCUSIÓN

Este estudio confirma la presencia de *aac(6’)-Ian* en el 15,5% (13/84) de los aislados de *K. pneumoniae* y *E. coli* con sensibilidad reducida a amikacina, asociado a genes bla_NDM_, *bla*_*PER-2*_ y *qnrVC1*. Estos determinantes se localizan en plásmidos IncC2 de alta transferibilidad, cuya estructura genómica mantiene una identidad superior al 95% con el plásmido *pEcCol16ndm* detectado previamente en Perú ^8^, demostrando la persistencia de una plataforma de multirresistencia en la región.

La coexistencia de *bla*_*NDM*_ *, bla*_*PER-2*_ *y qnrVC1* previamente descrita en Perú en un replicón IncC2 ^8^ fue igualmente identificada en las cepas silvestres y en sus transconjugantes, lo que explica la resistencia a amikacina, kanamicina, y sulfametoxazol, asociada a resistencia a β-lactámicos, inhibidores de β-lactamasas y disminución de sensibilidad a quinolonas, sin afectar la actividad de la gentamicina.

Asimismo, el análisis de las 13 EPC confirmó la presencia del replicón IncC2. El gen *aac(6’)-Ian* ha sido previamente reportado en este tipo de plásmidos movilizables y se ha asociado con *bla*_*NDM-1,*_ *bla*_*CTX-M-15*_ *, bla*_*GES-2*_ *, bla*_*TEM-1A*_ *, bla*_*OXA-1*_ *, bla*_*ACT-15*_ *, aac(3’)-IIa*^12^*, así como con bla*_*TLA-3*_ *, bla*_*TEM*_ y *aac(3’)-II*^7^.

Corroboramos estos hallazgos mediante la secuenciación genómica de seis cepas portadoras de *aac(6’)-Ian* seleccionadas por conveniencia. Gonzales Escalante reportó una configuración genética en torno a *aac(6’)-Ian* que incluía *qnrVC1*, *bla*_*NDM*_ *y bla*_*PER-2*_ , incrustados en las islas de resistencia ARI-B en una cepa de *E. coli*^8^. Además, el análisis de alineamiento con NZ_JANIDU010000010.1 mostró una amplia cobertura de secuencia, lo que respalda la hipótesis de que el plásmido IncC2 ha estado circulando en el entorno local por al menos cinco años ^8^. La presencia del tipo IncC2 diseminado en diversos ST nos permite inferir su propagación exitosa.

La actividad de plazomicina no se vio afectada por la presencia de *aac(6’)-Ian*, al igual que gentamicina, esto sugiere que ambas podrían constituir opciones terapéuticas frente a EPC productoras de esta enzima. La preservación de la actividad de plazomicina podría explicarse por diferencias estructurales, como el grupo 2-hidroxietilo en la posición 6’, que le permitiría evadir este mecanismo de resistencia ^13^. Aunque estos resultados *in vitro* son alentadores, su implementación clínica dependerá de su disponibilidad en escenarios locales, por lo que se requieren estudios adicionales que consoliden su utilidad frente a cepas multirresistentes asociadas a carbapenemasas.

En Latinoamérica se ha documentado la circulación de plásmidos que acumulan múltiples determinantes de resistencia. Millán *et al*. demostraron la movilización de *bla*_*CTX-M*_ asociada a ISCR1 en plásmidos IncN e IncFIIA en Venezuela, mientras que reportes regionales evidencian la co-circulación de BLEE y carbapenemasas en entornos hospitalarios ^14^. Recientemente, Cordeiro *et al*. describieron *bla*_*NDM-1*_ en un plásmido IncC2 en Uruguay, destacando la emergencia hemisférica de esta plataforma^15^. En este contexto, la asociación de *bla*_*NDM*_ con IncC2 observada en nuestro estudio se integra en un patrón regional de evolución plasmídica multirresistente que limita las opciones terapéuticas y refuerza la necesidad de fortalecer la vigilancia genómica.

La creciente detección regional de mecanismos como el descrito en este estudio refuerza la necesidad de estandarizar la secuenciación genómica en el marco de GLASS (Global Antimicrobial Resistance Surveillance System) ^16^, incorporando herramientas moleculares a la vigilancia de patógenos prioritarios. Esto fortalecería la toma de decisiones clínicas y la integración de Latinoamérica en redes globales de monitoreo de resistencia. A nivel nacional, la articulación de estas estrategias con los programas de optimización del uso de antimicrobianos (PROA) es fundamental para limitar la expansión de plataformas plasmídicas emergentes como IncC2 ^17^.

Este estudio presenta limitaciones que deben reconocerse. Los aislamientos proceden de un único laboratorio privado, lo que puede afectar su representatividad nacional, y no se contó con información clínica detallada. Además, 13 de 84 cepas fueron positivas para *aac(6’)-Ian* y solo seis se secuenciaron, lo que restringe la generalización estructural del plásmido. No obstante, estos aspectos no disminuyen la relevancia epidemiológica de los hallazgos.

En conclusión, reportamos la presencia de *aac(6’)-Ian* en 13 aislamientos de EPC resistentes a amikacina portadores de *bla*_*PER-2*_ *, bla*_*NDM*_ y *qnrVC1* en plásmidos IncC2. Estos hallazgos indican el riesgo de diseminación de la resistencia entre especies bacterianas, reduciendo las opciones terapéuticas para ITU complicadas debido a su potencial de diseminación horizontal.
